# Gain of function AMP‐activated protein kinase *γ*3 mutation (AMPK *γ*3^R200Q^) in pig muscle increases glycogen storage regardless of AMPK activation

**DOI:** 10.14814/phy2.12802

**Published:** 2016-06-14

**Authors:** Tracy L. Scheffler, Sungkwon Park, Peter J. Roach, David E. Gerrard

**Affiliations:** ^1^ Department of Animal and Poultry Sciences, Litton‐Reaves HallVirginia TechBlacksburgVirginia; ^2^ Department of Biochemistry and Molecular BiologyIndiana University School of MedicineIndianapolisIndiana; ^3^ Present address: Department of Animal SciencesUniversity of FloridaGainesvilleFlorida32611‐0910; ^4^ Present address: Sejong UniversityFaculty of Food Science and TechnologySeoulSouth Korea

**Keywords:** Calcium, glucose 6 phosphate, glycogen synthase, skeletal muscle, UDP‐glucose pyrophosphorylase

## Abstract

Chronic activation of AMP‐activated protein kinase (AMPK) increases glycogen content in skeletal muscle. Previously, we demonstrated that a mutation in the ryanodine receptor (RyR1^R615C^) blunts AMPK phosphorylation in longissimus muscle of pigs with a gain of function mutation in the AMPK *γ*3 subunit (AMPK *γ*3^R200Q^); this may decrease the glycogen storage capacity of AMPK *γ*3^R200Q^ + RyR1^R615C^ muscle. Therefore, our aim in this study was to utilize our pig model to understand how AMPK *γ*3^R200Q^ and AMPK activation contribute to glycogen storage and metabolism in muscle. We selected and bred pigs in order to generate offspring with naturally occurring AMPK *γ*3^R200Q^, RyR1^R615C^, and AMPK *γ*3^R200Q^ + RyR1^R615C^ mutations, and also retained wild‐type littermates (control). We assessed glycogen content and parameters of glycogen metabolism in longissimus muscle. Regardless of RyR1^R615C^, AMPK *γ*3^R200Q^ increased the glycogen content by approximately 70%. Activity of glycogen synthase (GS) without the allosteric activator glucose 6‐phosphate (G6P) was decreased in AMPK *γ*3^R200Q^ relative to all other genotypes, whereas both AMPK *γ*3^R200Q^ and AMPK *γ*3^R200Q^ + RyR1^R615C^ muscle exhibited increased GS activity with G6P. Increased activity of GS with G6P was not associated with increased abundance of GS or hexokinase 2. However, AMPK *γ*3^R200Q^ enhanced UDP‐glucose pyrophosphorylase 2 (UGP2) expression approximately threefold. Although UGP2 is not generally considered a rate‐limiting enzyme for glycogen synthesis, our model suggests that UGP2 plays an important role in increasing flux to glycogen synthase. Moreover, we have shown that the capacity for glycogen storage is more closely related to the AMPK *γ*3^R200Q^ mutation than activity.

## Introduction

AMP‐activated protein kinase (AMPK) plays a key role in cellular energy homeostasis in skeletal muscle. AMPK is a heterotrimeric serine/threonine kinase composed of a catalytic *α* and regulatory *β* and *γ* subunits. Decreasing energy charge (ATP:AMP) enhances AMPK activation by allosteric binding of AMP to the *γ* subunit, and promotes phosphorylation of the activating site, *α*Thr‐172, by upstream kinases (Stein et al. 2000; Suter et al. 2006; Gowans et al. 2013). In order to preserve cellular ATP, activated AMPK acutely inhibits anabolic pathways and stimulates catabolic pathways involved in carbohydrate, lipid, and fatty acid metabolism. AMPK also modulates long‐term adaptation by coordinating changes in gene and protein expression.

The *γ*3 subunit is highly expressed in glycolytic skeletal muscle and plays a key role in adaptation and fuel metabolism (Mahlapuu et al. 2004). Activating mutations in *γ*3 contribute to significant elevations in glycogen content in glycolytic muscles of mice (Barnes et al. 2004), pigs (Milan et al. 2000), and humans (Costford et al. 2007). Glycogen synthesis is primarily controlled by glucose transport and glycogen synthase (GS) activity (Azpiazu et al. 2000). AMPK facilitates contraction‐induced, insulin‐independent glucose uptake by promoting translocation of the muscle‐specific glucose transporter (GLUT4) to the cell membrane (Kurth‐Kraczek et al. 1999; Jørgensen et al. 2004b). Moreover, chronic activation of AMPK results in adaptive changes in glucose metabolism, including increased GLUT4 protein content (Holmes et al. 1999). However, AMPK*γ*3 knockout mice exhibit normal glucose tolerance and similar muscle glycogen content as wild type, but show reduced capacity to resynthesize glycogen after exercise (Barnes et al. 2004). Together, this suggests that glucose transport is not the primary step contributing to *γ*3‐mediated glycogen synthesis; instead, glycogen synthase activity or intermediary steps may limit storage of glucose as glycogen.

The regulation of GS is rather complex; it is negatively regulated by phosphorylation by AMPK (Jensen et al. 2006) as well as several other protein kinases. Although nine phosphorylation sites have been identified, only four sites (2, 2a, 3a, 3b) are considered the most influential for regulating GS activity in muscle (Roach et al. 2012). However, glucose 6‐phosphate (G6P) can completely overcome the inhibitory effects of phosphorylation, and thus restore full activity of GS (Roach et al. 2012). In this manner, limiting glucose transport restricts G6P available for glycolysis and glycogen synthesis, whereas higher rates of glucose transport enhance G6P levels and flux toward glycogen synthesis. In fact, Hunter et al. (Hunter et al. 2011) demonstrated that the primary molecular mechanism by which AMPK enhances glycogen storage despite GS phosphorylation is via G6P‐induced allosteric activation. Chronic activation of AMPK likely enhances G6P content by increasing hexokinase content (Leick et al. 2010) and activity (Holmes et al. 1999; Granlund et al. 2011).

Pigs with a single‐nucleotide polymorphism in the regulatory *γ*3 subunit of AMPK possess increased glycogen content in white skeletal muscle (Milan et al. 2000). This polymorphism results in an amino acid substitution (R200Q) in domains involved in binding AMP or ATP; ultimately, the AMPK*γ*3^R200Q^ mutation results in lack of AMP dependence and elevated basal activity of AMPK (Barnes et al. 2004). However, we have shown that phosphorylation of AMPK, as well as GLUT4 protein content, are blunted in AMPK*γ*3^R200Q^ pig muscle that also has a mutation in ryanodine receptor 1 (RyR1^R615C^), or the calcium release channel (Park et al. 2009). Thus, we anticipated that blunted AMPK phosphorylation and GLUT4 protein content in AMPK*γ*3^R200Q^ + RyR1^R615C^ muscle may also blunt hexokinase‐mediated increases in G6P and glycogen synthase activity, and thus limit glycogen storage compared to AMPK*γ*3^R200Q^_._ Yet, the mitochondrial content and oxidative capacity of AMPK*γ*3^R200Q^ + RyR1^R615C^ is increased and similar to AMPK*γ*3^R200Q^ muscle, suggesting that the mutation is sufficient to alter fuel storage and utilization (Scheffler et al. 2014). Thus, our objective was to use our AMPK and RyR1 pig model to understand how AMPK*γ*3^R200Q^ and AMPK activation contribute to glycogen storage and metabolism in muscle.

## Materials and Methods

### Animals

Animals were bred and reared at the Purdue University Swine Center and the Virginia Tech Swine Center, and all procedures were carried out in accordance with the guidelines of each university's Institutional Animal Care and Use Committee. Pigs heterozygous at the RyR1 and AMPK*γ*3 loci were bred to generate all possible genotype combinations. Female and castrated male pigs were reared under standard conditions and fed ad libitum. At approximately 120 kg, animals were transported to the university's meat science center and harvested. Immediately after exsanguination, muscle samples (~5–10 g) were collected from the lumbar region of the longissimus muscle. Samples were immediately frozen in liquid nitrogen and stored at −80°C until further analysis.

### Genotype determination

Genotypes were determined using polymerase chain reaction (PCR) restriction fragment length polymorphism technique. DNA was isolated from blood or tissue and used for PCR amplification. PCR products were digested with appropriate restriction enzyme overnight and fragments were separated on an agarose gel stained with ethidium bromide for visualization. For determination of AMPK*γ*3 genotype, the primers were (5′–3′) AAATGTGCAGACAAGGATCTC (forward) and CCCACGAAGCTCTGCTT (reverse). AMPK*γ*3 products were digested with restriction enzyme BsrBI. Pigs were evaluated for RyR1 genotype (Fujii et al. 1991) following the procedures outlined by O'Brien et al. (O'Brien et al. 1993). Those that were homozygous “normal” (wild type) at both RyR1 and AMPK loci were considered control while those pigs designated RyR1^R615C^ were homozygous mutant. RyR1 heterozygotes were excluded because they tend to exhibit an intermediate phenotype. In contrast, AMPK*γ*3 mutation is dominant, so both homozygous mutant and heterozygotes were utilized (designated AMPK*γ*3^R200Q^). Finally, pigs denoted as AMPK*γ*3 + RyR1 mutants were either heterozygous or homozygous mutant at AMPK*γ*3 locus, and homozygous mutant at the RyR1 locus.

### Glycogen synthase and phosphorylase activity

Enzyme activities were determined in muscle homogenates. Muscles were powdered in liquid nitrogen and homogenized on ice in buffer containing protease and phosphatase inhibitors (50 mmol/L Tris pH 7.8, 10 mmol/L EDTA, 10 mmol/L EGTA, 100 mmol/L NaF, 35 mg/mL tosyllysine chloromethyl ketone hydrochloride, 2 mmol/L benzamidine, 10 mg/mL leupeptin, and 0.5 mmol/L *β*‐mercapthoethanol). Glycogen synthase activity was quantified by the incorporation of [U‐^14^C]glucose from UDP[U‐^14^C]glucose into glycogen. The assay was conducted in the absence or presence of 10 mmol/L G6P (−G6P or +G6P), and activity ratio (−G6P/+G6P) was also determined. Glycogen phosphorylase activity was measured in the direction of glycogen synthesis from [U‐^14^C]glucose 1 phosphate (G1P) in the absence or presence of 3 mmol/L 5′AMP. Glycogen phosphorylase activity ratio was determined by dividing activity without AMP by activity with AMP (−AMP/+AMP).

### Glycogen

Glycogen content was determined as previously described (Scheffler et al. 2013). Briefly, frozen muscle was powdered in liquid nitrogen and subjected to acid hydrolysis at 95°C. Another portion of frozen muscle was homogenized in perchloric acid, centrifuged, and the resulting supernatant was used for glucose, glucose‐6‐phosphate, and lactate analysis. Muscle glucose (free glucose and glucose resulting from glycogen hydrolysis), glucose‐6‐phosphate, and lactate concentrations were determined using enzyme analytical methods (Bergmeyer 1974). These metabolite concentrations were used to calculate total glycogen content (in glucose equivalents) using the formula: glycogen = glucose + G6P + (lactate/2).

### Western blotting

Procedures for sample processing were determined based on preliminary testing. For detection of hexokinase 2 (HK2), UGP2, and GS, muscle samples were homogenized and sonicated in HEPES buffer (25 mmol/L HEPES, 1 mmol/L EDTA, 1 mmol/L benzamidine, 1 mmol/L 4‐(2‐aminoethyl)‐benzene + sulfonyl fluoride, 1 μmol/L aprotinin, 1 μmol/L leupeptin, 1 μmol/L pepstatin, pH 7.5) and retained as a total homogenate. Protein concentration was determined using BCA protein assay kit (Pierce, Rockford, IL) and samples were diluted to yield equal protein concentration and mixed with Laemmli buffer. Protein samples were separated using SDS‐PAGE and transferred to nitrocellulose membranes. Membranes were blocked in StartingBlock Blocking Buffer (Thermo Scientific). Blots were probed with primary antibody (UGP2 and HK2, Sigma, St. Louis, MO) and GS (Bioss; Woburn, MA) prepared in blocking buffer with 0.05% tween and washed in Tris‐buffered saline with 0.05% tween. Then, blots were incubated with the appropriate IRDye^®^ 680 or 800 conjugated anti‐IgG antibody (LI‐COR^®^ Biosciences, Lincoln, NE). Bands were visualized using Odyssey ^®^ Infrared Imaging System (LI‐COR^®^ Biosciences) and quantified using the manufacturer's software.

### Statistical analysis

Data were analyzed using SAS‐JMP (Statistical Analysis Software; Cary, NC). The model was a two‐way ANOVA with main effects of AMPK (wild type or mutated) and RyR1 (wild type or mutated) genotypes and their interaction (AMPK*RyR1). Data are represented as LSM ± SE. If the interaction was significant, differences between genotype combinations were determined using Tukey's adjustment for multiple comparisons. *P* < 0.05 was considered significant.

## Results

Previously, we reported that pigs with both AMPK and RyR1 mutations have blunted AMPK phosphorylation and decreased GLUT4 protein expression compared to pigs possessing only AMPK mutation (Park et al. 2009). Yet, despite these differences, AMPK + RyR1 mutant muscle exhibits some traits associated with AMPK activation, such as increased oxidative capacity (Scheffler et al. 2014). Therefore, our goal was to understand the contribution of AMPK*γ*3^R200Q^ and AMPK activation to glycogen storage and metabolism in pig longissimus muscle.

### Muscle glycogen (sum of equivalents), glycogen, G6P, and lactate

**Figure 1:**
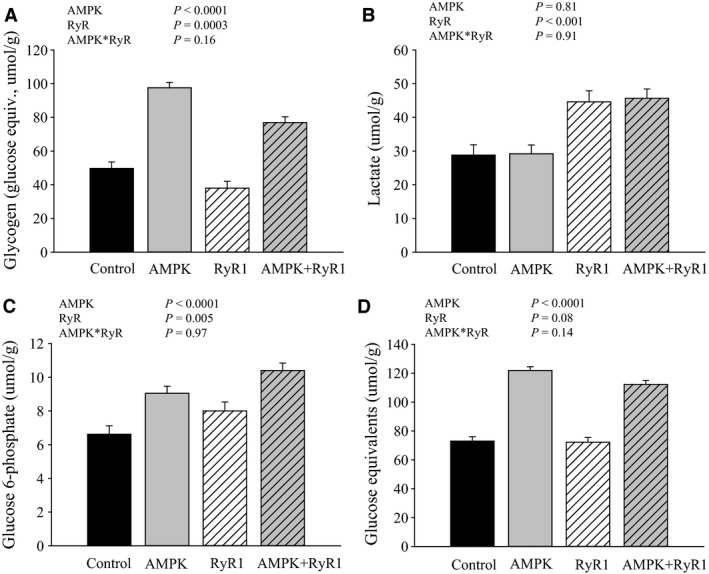
Influence of AMPK and RyR1 mutations on glycogen (A); glucose 6‐phosphate (B); lactate (C); and glucose equivalents (D) in longissimus muscle. (all are expressed in *μ*mol/g wet weight). Glucose equivalents was calculated as Gly + Glc + G6P + 1/2lactate; or ½ GP. Data are LSM ± SE. (*n* = 7–9 pigs per genotype).

AMPK genotype influenced (*P* < 0.0001) glycogen content in longissimus muscle (Fig. 1A); In contrast, RyR1^R615C^ decreased glycogen (*P* = 0.0003), consistent with increased lactate content (Fig. 1B, *P* < 0.001), possibly linked to increased phosphorylase activation (see below). In addition, both AMPK and RyR mutations influenced G6P levels (*P* < 0.0001 and *P* = 0.005, respectively). To better reflect total initial glycogen levels in muscle, we calculated the sum of major glycolytic metabolites (glucose equivalents, Fig. 1D). AMPK*γ*3^R00Q^ muscle had approximately 70% greater glucose equivalents, regardless of RyR genotype. RyR1^R615C^ genotype tended to decrease glycogen (*P* = 0.08), but the change was relatively small (~5%).

### Glycogen synthase activity and content

**Figure 2:**
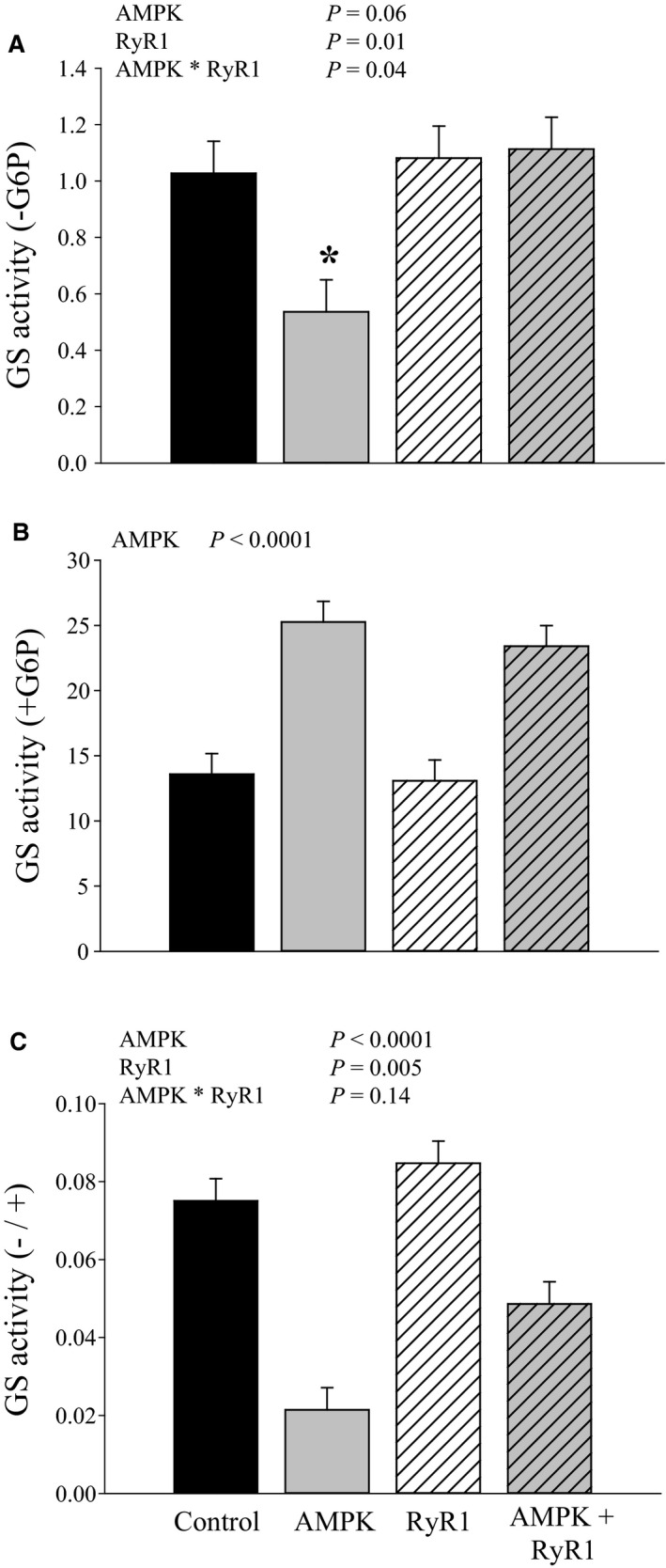
Glycogen synthase activity in AMPK and RyR genotypes. (A) Glycogen synthase activity in the absence of glucose 6‐phosphate. (B) Glycogen synthase activity in the presence of glucose 6‐phosphate. (C) Activity of glycogen synthase expressed as ratio (−/+ G6P). Data are LSM ± SE (*n* = 5 pigs per genotype). *indicates significantly different than other genotypes (*P* < 0.05).

Glycogen synthase is considered to be the rate‐limiting enzyme for glycogen synthesis. Phosphorylation contributes to down‐regulation of GS activity, but inactivation can be completely overcome by increasing the allosteric activator, G6P. Thus, GS is typically assayed in the absence and presence of G6P, and the resulting ratio (−/+ G6P) is used as an indication of the phosphorylation state of GS. An AMPK × RyR1 genotype interaction (*P* = 0.04) influenced GS activation in the absence of G6P, as demonstrated by the lower GS activity (*P* < 0.05) in AMPK*γ*3^R200Q^ muscle relative to all other genotypes (Fig. 2A). However, G6P increased (*P* < 0.0001) GS activity about twofold in muscle with AMPK*γ*3^R200Q^, regardless of the RyR1 mutation. For GS activity ratios, AMPK*γ*3^R200Q^ muscle exhibited the lowest activity, whereas AMPK*γ*3^R200Q^ + RyR1^R615C^ was intermediate, and control and RyR1^R615C^ were the highest. The low activity ratio of AMPK*γ*3^R200Q^ muscle reflects both inactivation of GS by phosphorylation in the absence of G6P and increased activity in the presence of G6P, whereas the intermediate value of AMPK*γ*3^R200Q^ + RyR1^R615C^ muscle is due exclusively to increased activity with G6P.

**Figure 3:**
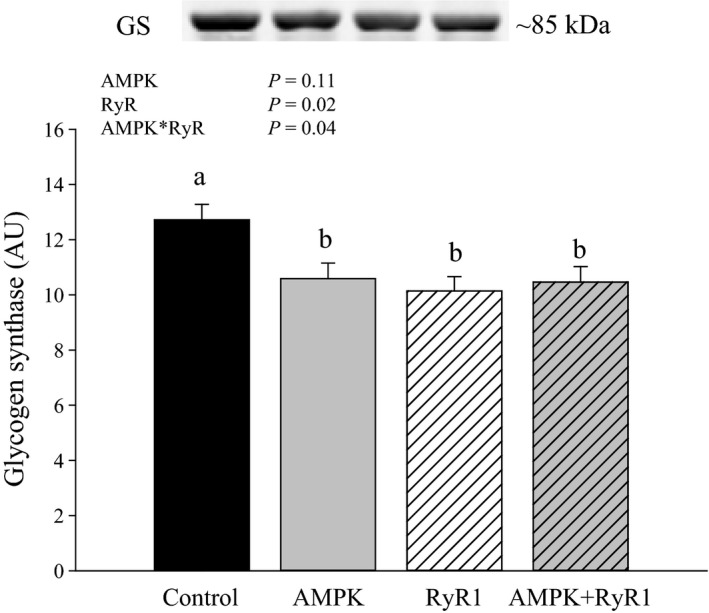
Glycogen synthase protein content of AMPK and RyR genotypes. Data are LSM ± SE (*n* = 6–7 pigs per genotype). ^a,b^Genotypes not sharing a common superscript are significantly different (*P* < 0.05).

Because total activity (+G6P) is often related to protein content (Manchester et al. 1996), we evaluated GS protein content by western blot. Unexpectedly, GS content was about 20% higher (*P* < 0.05) in wild‐type pigs relative to all other genotypes (Fig. 3).

### Enzymes in glycogen synthesis pathway

**Figure 4:**
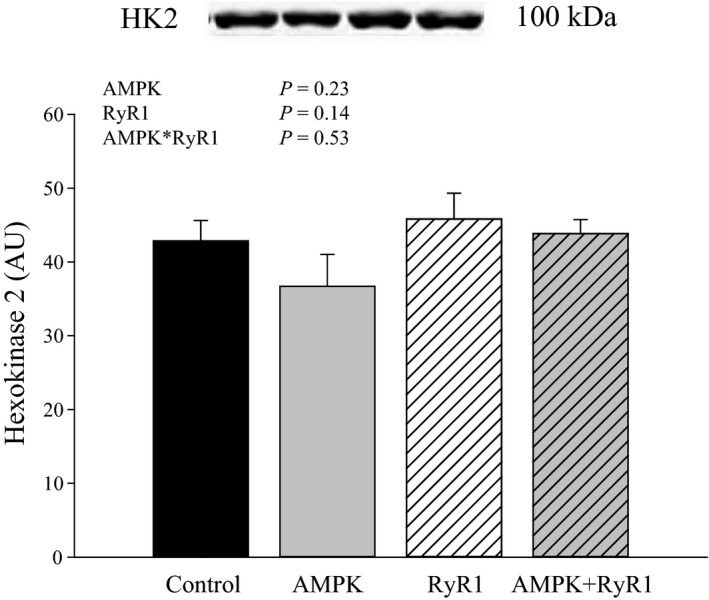
Hexokinase 2 protein content of AMPK and RyR genotypes. Data are LSM ± SE (*n* = 6–7 pigs per genotype).

**Figure 5:**
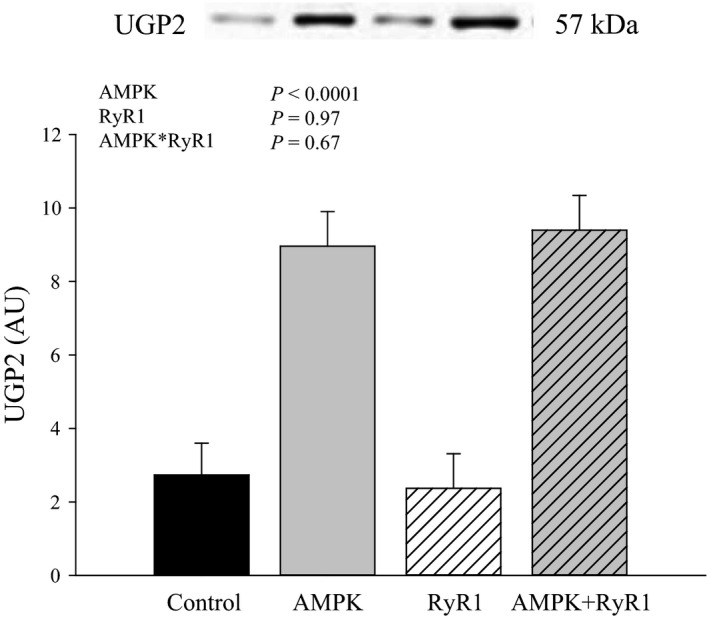
UDP‐glucose pyrophosphorylase 2 protein content of AMPK and RyR genotypes. Data are LSM ± SE (*n* = 6–7 pigs per genotype).

Although GS is considered the rate‐limiting enzyme, glycogen synthesis rate is dependent on glucose supply. The first step is the uptake of glucose into the cell via glucose transporters. Previously, we determined that GLUT4 protein content in muscle with AMPK*γ*3^R200Q^ was enhanced relative to control, whereas GLUT4 in AMPK*γ*3^R200Q^ + RyR1^R615C^ muscle was similar to control (Park et al. 2009). Enzymes downstream of glucose entry are HK2, phosphoglucomutase, and UGP2. Hexokinase 2 protein content was not affected by genotype (Fig. 4). However, AMPK*γ*3^R200Q^ increased (*P* < 0.0001) the content of UGP2 approximately threefold (Fig. 5).

### Glycogen phosphorylase

**Figure 6:**
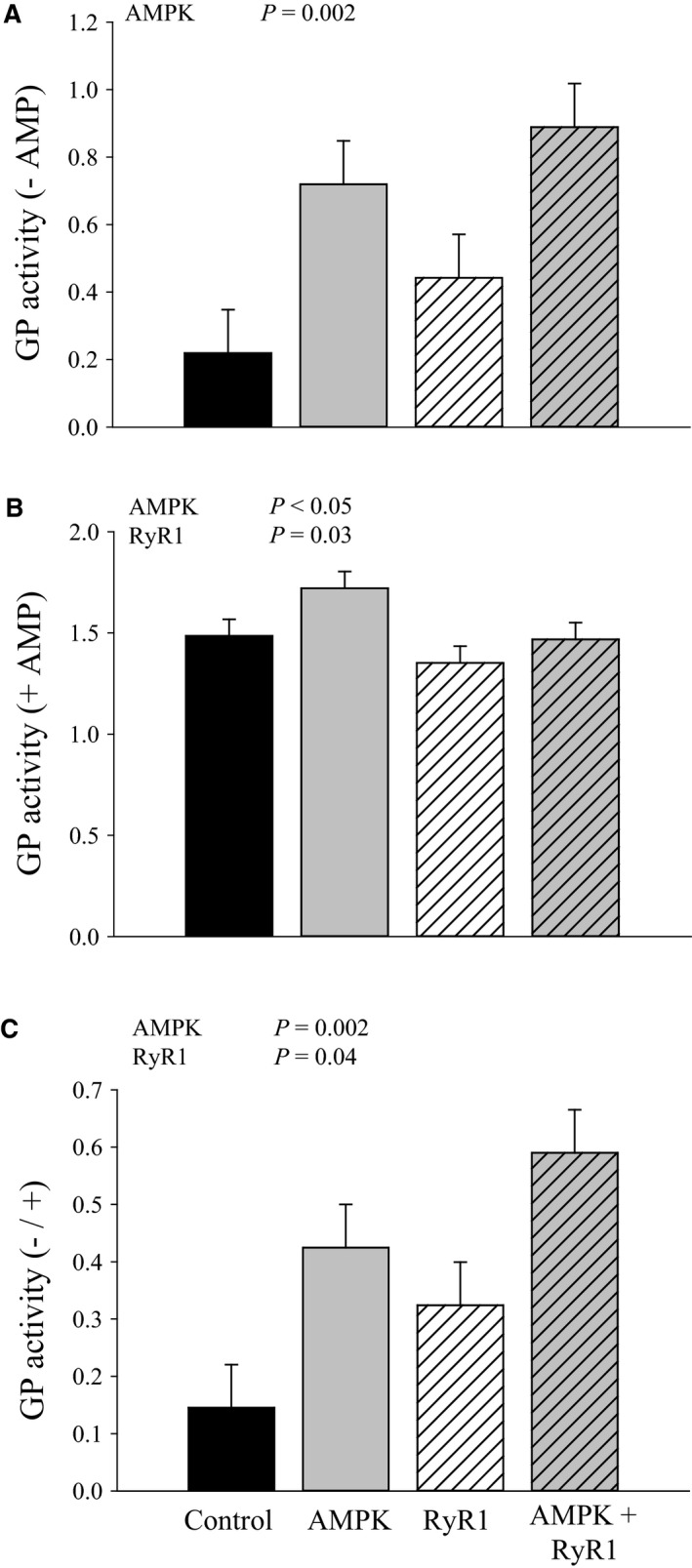
Glycogen phosphorylase activity in AMPK and RyR genotypes. (A) Glycogen phosphorylase activity in the absence of AMP. (B) Glycogen synthase activity in the presence of AMP. (C) Activity of glycogen phosphorylase expressed as ratio (−/+ AMP). Data are LSM ± SE (*n* = 5 pigs per genotype).

Since glycogen content is the net result of glycogen synthesis and degradation, we evaluated the activity of glycogen phosphorylase (GP), the rate‐limiting enzyme for glycogen breakdown. In the absence of AMP, the AMPK mutation contributed to increased GP activity (*P* = 0.002; Fig. 6), suggesting greater phosphorylation (activation) of GP in muscle with AMPK*γ*3^R200Q^. AMPK and RyR1 genotype also exerted significant (*P* < 0.05 and *P* = 0.03, respectively), but rather small effects on GP activity (+AMP). Although AMPK*γ*3^R200Q^ was associated with increased activity, RyR1^R615C^ decreased GP (+AMP) activity. Therefore, the GP activity ratio (−/+AMP) was lowest in control and highest in AMPK*γ*3^R200Q^ + RyR1^R615C^ muscle.

## Discussion

AMPK signaling plays important roles in acute and adaptive regulation of muscle metabolism. Under acute energy stress, AMPK is well‐documented to protect cellular energy levels by down‐regulating anabolic pathways, such as glycogen synthesis. Yet, AMPK's role in limiting glycogen synthase activity is not consistent with the dramatic increase in muscle glycogen in pigs and mice with an AMPK‐activating mutation. Increases in muscle glycogen despite inactivation of GS are largely mediated by allosteric activation of GS by G6P (Bouskila et al. 2010; Hunter et al. 2011). Enhanced glucose transport contributes to elevated G6P, which overcomes the inhibitory effect of phosphorylation on glycogen synthase, and thus promotes glycogen storage (Hunter et al. 2011). However, we previously demonstrated that pigs with AMPK*γ*3^R200Q^ exhibit enhanced AMPK activity and a concordant increase in GLUT4 content, whereas these effects are blunted in muscle with AMPK*γ*3^R200Q^ + RyR1^R615C^ (Park et al. 2009). In turn, we expected these differences in AMPK activity and GLUT4 content would be associated with altered glycogen storage capacity and metabolism.

Curiously, AMPK*γ*3^R200Q^ + RyR1^R615C^ muscle contains similar glycogen as AMPK*γ*3^R200Q^ muscle, which is approximately 70% greater than wild‐type and RyR1^R615C^ littermates. In muscle, control of glycogen synthesis is distributed primarily between glucose transport and glycogen synthase (Azpiazu et al. 2000). GLUT4 content and translocation contribute significantly to glucose uptake into the cell, and initially, these steps were considered rate‐limiting for glycogen synthesis (Ren et al. 1993). Consistent with this concept, transgenic overexpression of GLUT4 in muscle increases glucose transport activity and enhances glycogen storage, without affecting activation of glycogen synthase (Hansen et al. 1995). However, GLUT4 knockout mice also possess greater muscle glycogen in the fasted state despite markedly reduced glucose transport (Kim et al. 2005), suggesting that glucose transport is not always rate‐limiting for glycogen synthesis. Along these lines, RyR1^R615C^ blunted AMPK*γ*3^R200Q^ ‐induced increases in GLUT4 content (Park et al. 2009), but did not diminish glycogen storage. Although GLUT4 content significantly contributes to glucose transport, other mechanisms may permit glycogen storage in AMPK*γ*3^R200Q^ + RyR1^R615C^ muscle.

Glycogen synthase is considered the key regulatory enzyme in glycogen synthesis. Due to the complex regulation of GS, including multiple phosphorylation sites, GS activity is typically assayed in the absence and presence of G6P. GS activity (‐G6P) was reduced only in AMPK*γ*3^R200Q^ muscle, which is consistent with increased AMPK phosphorylation and activity in AMPK*γ*3^R200Q^ but not AMPK*γ*3^R200Q^ + RyR1^R615C^ muscle (Park et al. 2009), and with activated AMPK functioning as a glycogen synthase kinase (Jørgensen et al. 2004a). However, GS activity (+G6P) was nearly double in AMPK mutated muscle (AMPK*γ*3^R200Q^ and AMPK*γ*3^R200Q^ + RyR1^R615C^) compared to wild type and RyR1^R615C^. Activity with G6P, also referred to as total GS activity, is thought to parallel GS content. Increasing GS content is sufficient to augment muscle glycogen synthesis and glycogen level without altering glucose transport (Manchester et al. 1996). However, AMPK*γ*3^R200Q^ did not increase GS protein content, as evidenced by western blot quantification. Similarly, Yu et al. (Yu et al. 2006) observed that muscles from AMPK*γ*3^R225Q^ mice exhibit increased GS activity compared to wild type, but do not have greater GS protein content. The disconnect between GS activity in the presence of G6P and GS protein may reflect an as yet unappreciated, stable mechanism to modify the enzyme activity. It is also possible that other mechanisms may make GS more sensitive to G6P in AMPK*γ*3^R200Q^ and AMPK*γ*3^R200Q^ + RyR1^R615C^ muscle.

Allosteric regulation of GS, and hence increased total GS activity, is the primary mechanism by which AMPK (Hunter et al. 2011) and insulin (Bouskila et al. 2010) promote glycogen accumulation. The increased flux through glucose transporters and HK2 contributes to elevated G6P and allosteric activation of GS. The AMPK and RyR mutations increased G6P levels in muscle. Elevated G6P in RyR1^R615C^ muscle is likely due to increased glycolytic flux, which is also consistent with the elevated lactate levels observed in muscle; pigs with this mutation are susceptible to stress and rapid metabolism (MacLennan and Phillips 1993). On the other hand, increased G6P in AMPK pigs may be associated with greater glucose transport. Others have shown that chronic activation of AMPK increases GLUT4 and increased HK2 activity (Holmes et al. 1999; Granlund et al. 2011). In AMPK*γ*3^R200Q^ + RyR1^R615C^ muscle, neither GLUT4 (Park et al. 2009) nor HK2 content (this study) was different from control. We did not determine HK2 activity, although it is possible that AMPK*γ*3^R200Q^ enhances G6P levels by increasing HK2 activity but not content.

High GS activity ratio (−/+ G6P) indicates that dephosphorylated (active) GS predominates. In mice genetically engineered to have altered expression of GS or its phosphatase, there is a positive relationship between GS activity ratio and glycogen content (Roach 2002). However, in our model, high activity ratio is a poor predictor of glycogen content because both control and RyR1^R615C^ muscle exhibit the highest activity ratios, but contain less glycogen than AMPK*γ*3^R200Q^ and AMPK*γ*3^R200Q^ + RyR1^R615C^ muscle. The inverse relationship between GS activity ratio and glycogen content is consistent with other reports incorporating genetic (Yu et al. 2006), exercise (Manabe et al. 2013), and fasting/refeeding (Jensen et al. 2006) approaches. Depending on the model, changes in the GS activity ratios may result from more adaptive changes, such as GS protein content, or by acute mechanisms involving regulation of glycogen synthase. For example, exercise training in rats promotes glycogen storage and coincides with a decrease in GS activity ratio, which is due almost exclusively to elevated total GS activity and increases in GS content (Manabe et al. 2013). On the other hand, in rats exposed to a fasting/refeeding approach, GS activity ratio was inversely related to glycogen content, but there were no significant differences in GS content (Jensen et al. 2006).

Elevated glycogen content in AMPK*γ*3^R200Q^ and AMPK*γ*3^R200Q^ + RyR1^R615C^ muscle parallels total GS activity, suggesting that capacity to stimulate glycogen synthase plays a major role in glycogen storage in our model. In turn, glycogen synthase activity is also a function of the metabolites and enzymatic activity in the steps preceding glycogen synthesis. Traditionally, other enzymes in the glycogen synthesis pathway, including phosphoglucomutase and UGP2, are not considered rate‐limiting. Overexpression of UGP2 in muscle does not affect glycogen content, glucose tolerance, or insulin‐stimulated rates of glucose incorporation into glycogen (Reynolds et al. 2005). Yet, various genetic models used to manipulate glycogen content indicate a link between UGP2 expression and glycogen level. In models manipulating GS, glycogen content is inversely related to UGP2 expression; UGP2 mRNA is increased in GS knockout mice lacking muscle glycogen, but decreased in mice overexpressing glycogen synthase (Parker et al. 2006). On the other hand, in models that manipulate AMPK, glycogen is positively related to UGP2 expression. AMPK*γ*3^R225Q^ mice exhibit greater UGP2 expression and deposit more muscle glycogen, whereas AMPK*γ*3 knockout mice have decreased UGP2 expression and possess less muscle glycogen. Increased UGP2 protein content in AMPK*γ*3^R200Q^ pig muscle reported herein and elsewhere (Hedegaard et al. 2004) is consistent with gene expression patterns in AMPK mice. Interestingly, UGP2 levels are strongly upregulated in hearts of mice with a mutation in AMPK*γ*2, the predominant cardiac isoform (Luptak et al. 2007); and upregulation of UGP2 precedes glycogen accumulation. These authors suggested that UGP2 helps “pull” glucose toward glycogen in cardiac muscle with mutated *γ*2. The equilibrium of the phosphoglucomutase reaction (G6P ↔ glucose 1‐phosphate) favors G6P; however, increasing utilization of glucose 1‐phosphate by UGP2 would help promote flux toward glucose 1‐phosphate, and in turn, UDP‐glucose. Moreover, Manchester et al. (Manchester et al. 1996) showed that GS overexpression decreases UDP‐glucose concentrations and this lack of substrate likely restricts glycogen accumulation. Therefore, increasing UGP2 content in skeletal muscle may be an important means of enhancing flux to glycogen synthase and promoting glycogen accumulation in AMPK*γ*3^R200Q^ and AMPK*γ*3^R200Q^ + RyR1^R615C^ muscle under some conditions.

Glycogen content in muscle is the net result of glycogen synthesis and degradation. Although certain glycogen storage diseases are caused by defects in glycogen degradation and/or glycolysis, mutations in the *γ*3 and *γ*2 subunits of AMPK have not been linked to compromised glycogenolysis (Estrade et al. 1994; Luptak et al. 2007). In fact, elevated glycogen in AMPK*γ*3^R225Q^ mouse muscle enhances capacity for anaerobic exercise compared to wild type (Barnes et al. 2005). In our hands, AMPK*γ*3^R200Q^ increases glycogen phosphorylase activity; Granlund (Granlund et al. 2011) also reported elevated GP activity in longissimus muscle of AMPK*γ*3^R200Q^ pigs compared to wild type. Clearly, glycogen phosphorylase activity does not help explain elevated glycogen content in our pig model; however, it does indicate that glycogen turnover is increased. Manchester et al. (Manchester et al. 1996) speculated that glycogen content influences GS and GP levels, but other models are not consistent with this concept.

Altogether, we have shown that AMPK*γ*3^R200Q^ and AMPK*γ*3^R200Q^ + RyR1^R615C^ muscle exhibit differences in GLUT4 as well as AMPK and GS activation; however, both clearly possess elevated total GS activity and high muscle glycogen. Our model demonstrates that the capacity for glycogen storage is more closely related to the AMPK mutation than activity. Additionally, we show that UGP2, previously considered a minor player in glycogen storage pathway, may be an important contributor to glycogen storage by promoting flux toward glycogen synthase.

## Conflict of Interest

None declared.
